# Implementation matters: program impact pathway analysis of four sectoral nutrition-sensitive interventions in Anambra and Kebbi states, Nigeria

**DOI:** 10.1080/16549716.2025.2519677

**Published:** 2025-06-26

**Authors:** Oluchi Ezekannagha, Scott Drimie, Dieter Von Fintel, Busie Maziya-Dixon, Xikombiso Mbhenyane

**Affiliations:** a Division of Human Nutrition, Department of Global Health, Faculty of Medicine and Health Sciences, Stellenbosch University, Tygerberg, South Africa; b CGIAR System Organization, c/o Alliance of Bioversity and CIAT, Rome, Italy; c Department of Food and Nutrition, International Institute of Tropical Agriculture, Ibadan, Nigeria; d Department of Economics, Faculty of Economic and Management Sciences, Stellenbosch University, Stellenbosch, South Africa

**Keywords:** Nutrition-sensitive programs, implementation barriers, facilitators, dose-reach-fidelity-recruitment, community engagement, adaptive management, Nigeria

## Abstract

**Background:**

Undernutrition in early childhood can be reduced when large-scale, nutrition-sensitive programs are delivered with adequate dose, reach, fidelity, and recruitment.

**Objectives:**

This study (i) investigates the implementation and impact pathways of four nutrition-sensitive programs in Kebbi and Anambra states, Nigeria: Early Childhood Development Education (ECCDE), Environmental Sanitation, Skills Acquisition, and Agricultural Transformation Support Program (ATASP-1) and (ii) identifies cross-sector factors that enable or hinder effective dose, reach, fidelity, and recruitment.

**Methods:**

The study employs qualitative methods such as document reviews, in-depth interviews, and site observations to explore the complexity of program delivery and the contextual factors that influence its outcomes.

**Results:**

All four programs showed dose–reach–fidelity–recruitment gaps in varying degrees: irregular training and equipment delayed dose; rural and low-income communities were least reached; weak quality control cut fidelity; and recruitment seldom penetrated remote areas. Barriers across sectors included insufficient infrastructure, shortages of trained personnel, and bureaucratic funding delays. Programs with robust community engagement, active multi-stakeholder collaboration, timely resource flow, and short ‘reviewandadapt’ cycles (ATASP1 in both states; ECCDE in Anambra) overcame many shortfalls, whereas those lacking these features underperformed (Environmental Sanitation in Anambra; Skills Acquisition in Kebbi).

**Conclusion:**

Closing Nigeria’s nutrition-sensitive implementation gap demands a dual response: fix tangible barriers – staffing, infrastructure, and procurement – and institutionalize community-led planning and adaptive management to keep dose–reach–fidelity–recruitment on track. Doing so will improve program reach and quality and accelerate progress against child undernutrition.

## Paper Context

- **Main findings**: Gaps in dose, reach, fidelity, and recruitment were largest where resources and staff were limited, but programs that paired strong community engagement with rapid course correction achieved measurably better results.
- **Added knowledge**: By mapping these barriers and facilitators onto the dose–reach–fidelity–recruitment framework across four sectoral programs, the study pinpoints clear cross-sector entry points for nutrition-sensitive delivery – staffing, outreach design, and feedback loops.
- **Global health impact for policy action:** Improving frontline resources, institutionalizing community participation, and embedding short adaptive review cycles can make nutrition-sensitive programs more effective and equitable.

## Background

Despite steady progress, Nigeria still faces a high burden of child undernutrition, with stunting affecting more than one-third of under-fives [1]. Interventions that act on the basic and underlying determinants of nutrition – agriculture, WASH, education, and social protection – are therefore indispensable [2,3]. Evidence from multiple low‑ and middle‑income settings shows that nutrition‑sensitive programs often underdeliver because of implementation bottlenecks rather than weak theoretical logic [4,5]. Dose, reach, fidelity, and recruitment are the process measurements most frequently linked with impact, but how these dimensions falter varies by sectoral mandate and governance context [6–9]. Nigeria offers a case: the same federal structure devolves education, agriculture, environment, and social welfare functions to states that differ markedly in resources and administrative capacity.

This study uses insights from implementation research to evaluate Nigeria’s Early Childhood Development Education (ECCDE), Environmental Sanitation, Skills Acquisition, and Agricultural Transformation Agenda Support Program (ATASP-1) programs. The ATASP-1 initiative, in partnership with the African Development Bank, focuses on improving livelihoods and developing agricultural value chains in specific zones of Nigeria. The program targets key crops like rice, cassava, and sorghum. It aims to enhance rural infrastructures, such as schools, clinics, and roads, to stabilize rural communities and improve their quality of life. Its strategic activities are designed to fortify commodity value chains and elevate market access for farmers, safeguarding them from annual investment losses.

ECCDE provides educational programs for children under 5-years old within the public school system in Nigeria to impact optimal nutrition and overall child development. The program’s structure is designed to enhance children’s nutritional outcomes through quality education that includes age-appropriate training and resources. It emphasizes a multi-sectoral linkage, integrating education with nutrition, health, and social protection to support early childhood development comprehensively.

The Environmental Sanitation program, overseen by the Departments of Environmental Health in the state Ministries of Environment, aims to promote public health through improved environmental sanitation. It focuses on creating healthier living conditions by establishing sanitation task forces and enhancing waste management practices. The program also seeks to shape public attitudes toward environmental sanitation through Information, Education and Communication (IEC) materials and targeted awareness campaigns, particularly addressing waste disposal habits and malaria prevention.

Skills acquisition programs foster self-sufficiency among graduates by providing them with the necessary skills, resources, funding, and certification. These programs enhance participants’ livelihoods through vocational training that includes a component of nutritional knowledge, thereby enriching their health and food preparation skills. The aim is to equip participants with theoretical knowledge and practical skills that improve their employment prospects and productivity in the local economy.

Unpacking sector‑specific bottlenecks requires an analytical lens that goes beyond outputs. The PIP approach maps who does what, when, and under what assumptions, making it possible to locate break points along the pathway from activities to nutrition outcomes [10–13]. Coupled with qualitative process evaluation, PIP illuminates mechanisms that may generalize across contexts [14–16].

This study, therefore, asks two questions [1]: where along the dose–reach–fidelity–recruitment chain do implementation bottlenecks arise across four sectorally diverse, nutrition-sensitive programs in Nigeria? and [2] what barriers and facilitators – common or sector-specific – explain these bottlenecks?

## Methodology

### Study design

Utilizing a qualitative research design, this study employed document review, in-depth interviews, and site observations conducted in an ethnographic manner to assess the implementation of nutrition-sensitive programs, focusing on dose, reach, fidelity, and recruitment (Table 1).

**Table 1.** Process evaluation measures.

Dose	Number or frequency of program components delivered (e.g. training sessions, equipment) [17].
Reach	Number of people or target audience who participate or are directly reached by the program [17].
Fidelity	Quality and consistency with which the program is delivered as planned [18].
Recruitment	Methods and strategies used to attract and engage participants [17].

### Program selection

The research for this study was conducted in various state offices and program sites across the states of Anambra and Kebbi in Nigeria, selected for their diverse cultural and economic profiles. The study’s sampling process was carried out in several stages. Initially, programs overseen by national government ministries with high nutrition-sensitivity scores and nutrition-sensitive potential were selected. Programs were selected based on their potential for nutrition sensitivity and assessed using criteria such as impact on nutrition outcomes and scalability, as detailed in a forthcoming article. The ECCDE, ATASP-1, Environmental Sanitation, and Skills Acquisition programs were chosen from the Ministries of Education, Agriculture, Environment, and Social Welfare, respectively (Table 2).

**Table 2.** Summary of selected nutrition-sensitive programs.

	ATASP-1**	ECCDE*	Environmental Sanitation	Skills acquisition
Program description	An agricultural intervention program implemented in the states	The program refers to the education of children who are under 6-years old.	A sanitation program with a mandate to ensure optimal sanitation in every state.	A social welfare program that aims to provide diverse training for livelihoods.
Program period (Year of inception)	2014–2025	1999 as part of the Universal Basic Education Commission (UBEC)	1985, as part of the War Against Indiscipline	Actual year unknown. Implemented as part of the Ministry of Women’s Affairs
Regions	Selected states	National	National	National
Funding source	AfDB + State governments	State government	State government	State government
Sector	Agriculture	Education	WASH	Social Welfare
Pathway to optimal nutrition	Enhances agricultural livelihoods, rural infrastructure and develops value chains	Provides foundational education for children under 5; an avenue for growth, monitoring, and tracking	Improves public health through better waste management and environmental sanitation	Fosters self-sufficiency through vocational training; Nutrition knowledge classes
Core focus areas	Infrastructure, value chain enhancement	Education quality, child development	Waste disposal, public health improvement	Employment readiness, economic development

Source: Program and policy documents [19–21].

*Early Childhood Care and Development Education.

**ATASP-1: Agricultural Transformation Agenda Support Program-1.

### Selection of key program actors

This study did not evaluate the effectiveness of the programs but rather assessed the implementation and contexts underpinning these state-level programs. Key informants were chosen from the state, local government area, and community levels to provide their insights on how the programs were operationalized for impact. State program coordinators were responsible for selecting the communities and models to be visited. For adequate sampling, researchers ensured that each model sectoral site/community was in a different local government area.

**Figure 1.**
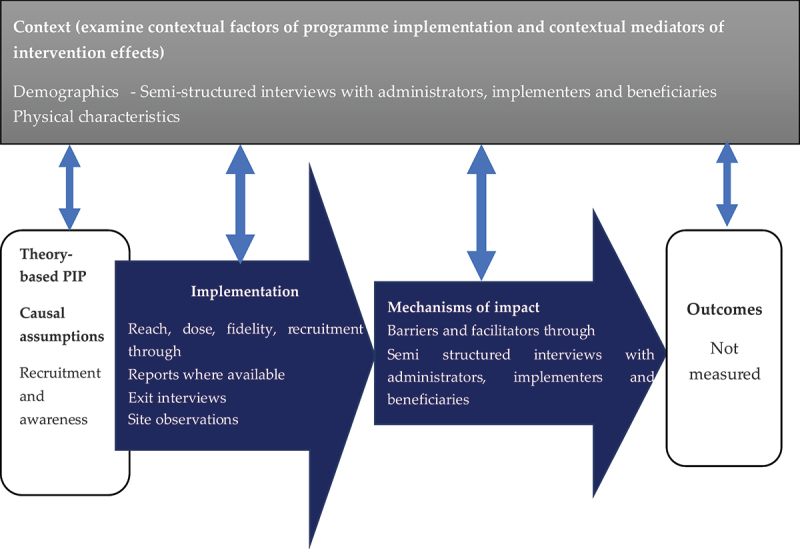
Data collection for process evaluation.

To ensure consistency in identifying participants, researchers created three categories: program administrators, program implementers, and program beneficiaries. This was necessary because different sectoral programs used varying titles for their staff. Program administrators, such as administrators, evaluators, managers, or directors of ministries, provide direction but are not involved in field operations, usually at the state level. Program implementers are the staff responsible for the daily activities of the programs at the program site, in most cases junior-mid level officers, while program beneficiaries are individuals who have or are still benefiting from the program in some way (Figure 1).

The data collection process involved document review, interviews, and site observations. Program documents were obtained from the state and Local Government Area (LGA) offices during site visits and interviews for document review. These documents included progress reports, final evaluation reports, informational pamphlets or booklets, and instructional and educational materials. The documents gathered detailed information on the program overview and its components. If these documents were unavailable, the researchers drew a program model based on their perceived implementation as described. These program models are not presented in this article.

To assess program delivery and impact in Nigeria, 24 informants were interviewed using a semi-structured interview guide. Fourteen interviews were conducted with program administrators at the state and local government area levels across various sectors. Eight interviews were conducted with program implementers, with four interviews conducted in each state. Additionally, exit interviews were conducted with at least two beneficiaries at each site to determine their exposure to the various programs. The sample size of 24 informants was determined based on saturation principles used in qualitative research, ensuring a diverse representation of perspectives while avoiding redundancy. Response rates were high, with over 90% of the contacted individuals agreeing to participate. Respondent characteristics, including their roles in the programs, are summarized in Table 3.

**Table 3.** List of interview participants.

Sector	Level	Anambra State	Kebbi state
		No of interviews	No of interviews
Agricultural Transformation Agenda Support Program 1	Program Administrator	1	1
	Program implementer	2	2
	Program beneficiary	2	5
Early Childhood Development Education	Program Administrator	2	2
	Program implementer	4	2
	Program beneficiary	2	2
Environmental Sanitation	Program Administrator	2	2
	Program Implementers	2	2
	Program beneficiary	2	2
Skills Acquisition	Program Administrator	2	2
	Program implementer	2	4
	Program beneficiary	3	4

All interviews were recorded, and participants were interviewed at their convenience. During the interviews, informants were asked about the program’s purpose, how the program activities work to achieve immediate and intermediate results related to learning, knowledge, and behavior change, and sector-specific outcomes. The interviews also explored facilitators, barriers to achieving outcomes, and factors influencing program implementation.

Since many of the local beneficiaries were rural community members who spoke Hausa and Igbo as their native languages and/or had limited understanding of technical or programmatic terminology, an interview guide using simple, everyday words was developed specifically for them. The guide was based on the English version of the interview guide, and back and front translation was employed to ensure accuracy. The English version of the semi-structured interview guide is provided as the supplementary material. This guide was developed to ensure clarity and consistency across interviews.

The researcher made observations and assessments using an observation guide during the site visits. Field notes were taken with detailed descriptions of activities. The observation visits were planned so that important events could be observed. The main objective was to gain a first-hand understanding and witness the implementation of any program activity. The observations were recorded using field notes during the activity. The researcher also wrote expanded notes immediately after each observation to describe the context and steps of the event, the beneficiaries’ responses, and the overall experience. The observation field notes were used to supplement the data collected through interviews.

### Data analysis

The lead author conducted the data analysis and reviewed it with co-authors to ensure analytical rigour and consistency. Each team member brought specialized expertise in qualitative data analysis, contributing to a thorough examination of the textual data and ensuring that interpretations were robust and well supported. The first step was recreating the program models and results frameworks based on program documents to understand the programs comprehensively. Interviews with program actors were also conducted to establish their organizational structures. The next step was to elaborate on the theories of change and program impact pathways using interview responses, program documents, and observation field notes.

MAXQDA software and manual notation were utilized to analyze the interview transcripts and identify predefined codes of program elements and emergent themes. These program elements included rationale, assumptions, inputs, activity process, outputs, impact process, outcomes, facilitator, barrier, and adaptation.

## Results

This study examined the impact of various nutrition-sensitive interventions within the unique socio-economic and cultural landscapes of Anambra and Kebbi states, Nigeria. Our primary objective was to understand the program design, implementation strategies, and the resulting outcomes, emphasizing ECCDE, Environmental Sanitation, Skills Acquisition, and the ATASP-1. By employing a qualitative methodology that included in-depth interviews, participant observation, and document analysis, we captured these interventions’ operational dynamics and impact pathways. The results first present findings on the operational domains – dose, reach, fidelity, and recruitment. Then, it follows with a synthesis of common facilitators and barriers. This approach allows us to delve into the structural and contextual factors that enabled or hindered achieving the desired outcomes. The results are organized by key implementation domains: dose, reach, fidelity, and recruitment, highlighting each program’s objectives, strategies, and observed outcomes in these areas.

The dose varied significantly across programs and states. In ECCDE, both Anambra and Kebbi faced challenges in consistently delivering essential components such as teacher training and classroom resources. Anambra partially compensated for this with community-driven initiatives, employing additional teachers at lower salaries. Environmental Sanitation showed a difference in doses, with Kebbi establishing a Sanitation Task Force to promote regular sanitation activities. In contrast, Anambra’s reliance on outsourced personnel created inconsistencies in service delivery. ATASP-1 struggled with the delayed distribution of imported agricultural equipment, affecting program delivery lightly, while Skills Acquisition programs varied in length and content, with Anambra offering longer, more practical training than Kebbi.

Several structural and contextual factors limited the reach. ECCDE faced significant reach challenges in rural areas, with Anambra’s program benefiting from parental involvement through the school boards, which boosted local enrollment. Environmental Sanitation programs in Kebbi reached a broader audience through targeted IEC materials and seasonal awareness campaigns. In contrast, Anambra’s reach was limited due to weaker governmental support and fewer community-based initiatives. ATASP-1 also encountered reach constraints; although Anambra managed to involve women’s seed transplanting groups, broader farmer engagement remained sporadic. Skills Acquisition programs aimed to reach unemployed youth and vulnerable populations but faced challenges in retaining participants, particularly in regions with limited follow-up support.

Fidelity emerged as a significant barrier to program effectiveness. In ECCDE, inconsistent quality assurance processes affected the fidelity of classroom instruction, particularly in Kebbi, where teacher training standards were not uniformly applied. Growth monitoring in ECCDE, critical for early nutritional assessment, was inadequately implemented across both states, with Anambra lacking systematic referral processes despite having basic equipment in schools. In environmental sanitation, fidelity issues include a lack of standardized training for health officers and insufficient resources for regular inspections, affecting sanitation quality. Fidelity in ATASP-1 was primarily consistent because it was implemented outside of the core Ministry of Agriculture by an ATASP-1 office. At the same time, Skills Acquisition programs suffered from inadequate entrepreneurial training, limiting participants’ ability to apply their skills sustainably.

ECCDE recruitment in Anambra was bolstered by community engagement and parental involvement. In Anambra, Environmental Sanitation efforts were compromised by a focus on revenue generation, which detracted from recruitment and engagement as a public health initiative. ATASP-1’s recruitment efforts were uneven; Anambra’s farmers benefitted from access to the federal Anchor Borrower Loan, yet gender-sensitive approaches were limited, affecting the engagement of youth and women in agriculture. Skills Acquisition recruitment targeted vulnerable groups but lacked active recruitment, limited participant retention, and long-term engagement.

Across all programs – ECCDE, Environmental Sanitation, Skills, Acquisition, and the ATASP-1 – several common facilitators and barriers emerged (See Table 4):

- Strong community engagement, stakeholder collaboration, and adequate resource allocation were pivotal in the successful execution and positive outcomes of programs.
- Insufficient infrastructure, lack of trained personnel, and bureaucratic inefficiencies stifled effective implementation.

**Table 4.** Comparative analysis of programs.

Programs (Sector)	ATASP-1 (Agriculture)	ECCDE (Education)	Environmental Sanitation (WASH)	Skills acquisition (Social Welfare and Labour)
Implementation	Delivered and utilized as planned in both states	Delivered as planned, suboptimal utilization in the Kebbi State	Weak delivery and utilization in the Anambra State	Partial implementation and utilization in both states
Community engagement	Strong in both states: participatory planning (Anambra) and the use of chieftaincy structures (Kebbi) raised the adoption of irrigation and seed packages.	High in Anambra (active parent committees); limited depth in Kebbi (attendance without voice).	Sporadic clean‑up days in Anambra; structured committees in Kebbi sustained hygiene practices.	Mobilization drives filled classes in Kebbi; virtually absent in Anambra, lowering uptake.
Multi‑stakeholder collaboration	Kebbi: tight gov’t – co‑operative links sped seed/fertilizer delivery. Anambra: coordination gaps slowed roll‑out.	Anambra: schools, NGOs and parents cocreated fundraisers and workshops. Kebbi: minimal joint action	Kebbi: environment ministry, NGOs, and CHWs ran integrated waste campaigns. Anambra: fragmented, short‑lived partnerships.	Anambra: training centres and industry codesigned curricula + apprenticeships; Kebbi: weak industry ties led to skills–job mismatch.
Resource allocation	Generally adequate but import/procurement delays.	Innovative community topups in Anambra; chronic shortfalls in Kebbi.	Kebbi taskforce model resourced; Anambra staffing and vehicle gaps.	Adequate training kits in Anambra; Kebbi expansion not funded (trainee numbers high without budget increase).
Infrastructure adequacy	Irrigation nets and feeder roads still lag, esp. remote Kebbi LGAs; storage delays in Anambra.	Classrooms/WASH facilities inadequate in both states, worse in Kebbi.	Waste‑disposal and recycling infrastructure scarce in Anambra; Kebbi maintenance underfunded.	Workshops and tools limited in both; Kebbi sites worse.
Skilled personnel	Extension cadre thin; few female agents in Kebbi (cultural constraint).	Teacher shortages and limited professional development; acute in Kebbi.	Too few trained environmentalhealth officers both states.	Trainer pool small; limited businessmentorship skills both states.
Bureaucratic efficiency	Moderate–high fund release and procurement lags.	Funding disbursement & teacher posting delays both states.	High redtape in Anambra; fewer bottlenecks in Kebbi.	Slow certification and monitoring cycles in both states.

### Strong community engagement

Strong community engagement played a crucial role in the success of ATASP-1 in both Anambra and Kebbi states, albeit in differing contexts and with varying degrees of impact. In Anambra, community engagement in ATASP-1 was highly effective, integrating local farmers into the decision-making process. Community meetings and participatory planning sessions were regularly held, allowing farmers to express their needs and preferences regarding agricultural support and infrastructure development. This engagement ensured that the interventions aligned with the farmers’ ‘ requirements, leading to higher adoption rates of new agricultural techniques and technologies introduced by the program. For instance, community-led discussions resulted in the prioritization of irrigation systems over other infrastructural developments, a critical need identified by the farmers due to the state’s lengthy dry season. This adaptive approach helped optimize resource utilization and improve the effectiveness of agricultural interventions. In Kebbi, community engagement in ATASP-1 leveraged local leadership and traditional structures to disseminate information and gather feedback. Regular involvement of local chieftains and agricultural leaders in program workshops and training sessions helped bridge the gap between government initiatives and community needs, facilitating broader acceptance and smoother implementation of new farming practices and systems. For example, introducing improved seed varieties and modern farming equipment was more readily accepted because the community leaders had been involved in the program’s early stages, ensuring that the local farming communities understood the benefits.

In Anambra State, the ECCDE program saw profound benefits from strong community engagement. Parents and local community leaders were involved in the school management and decision-making processes. For example, in several communities within Anambra, parents formed committees that worked closely with schools to organize fundraising events and educational workshops. These activities improved the schools’ physical infrastructure and enriched children’s academic content and engagement activities. The active involvement of parents led to increased enrollment rates and improved student attendance, as parents felt a direct stake in their children’s educational success. The engagement in Kebbi was less dynamic. While parents attended meetings and were physically present at school events, however, their involvement lacked depth regarding influence on educational policies or active participation in school governance. This resulted in a lesser sense of ownership and commitment to the ECCDE programs, reducing academic outcomes and limiting the program’s expansion.

Community engagement in environmental sanitation involved periodic community clean-up days in Anambra State that were well attended but lacked continuity and institutional support. The sporadic nature of these engagements led to temporary improvements in sanitation that were not sustainable in the long term. In contrast, Kebbi experienced more structured community engagement by establishing local sanitation committees that worked with local government and ward authorities. This sustained engagement helped implement more consistent sanitation practices, improving public health outcomes over extended periods. In Kebbi’s skills acquisition efforts, community engagement was focused on mobilizing attendees for the training, resulting in higher reach and participation in the program. In contrast, no community engagement occurred in Anambra, leading to reduced participation.

### Multistakeholder collaboration

The collaboration of ATASP-1 in Kebbi was particularly effective, involving local government and farmer cooperatives, leading to significant improvements in agricultural infrastructures, such as access roads, which directly benefited the local agricultural economy. For instance, the collaboration led to the streamlined delivery of high-quality seeds and fertilizers, increasing crop yields and enhancing market competitiveness. Moreover, regular collaborative meetings ensured that all parties were aligned with the program goals, leading to high efficiency in resource utilization. While ATASP-1 also involved multiple stakeholders in Anambra, it was marked by challenges in coordination, which slowed the momentum of project implementations. Issues such as delays in funding disbursements from government bodies and less frequent stakeholder meetings led to minor fragmentation in efforts.

Effective stakeholder collaboration in ECCDE includes engaging educational authorities, local communities, NGOs, and parents to enhance the quality and reach of educational programs. In Anambra, the ECCDE programs benefited from active community and parental involvement, highlighted by the formation of committees that worked closely with schools to organize fundraising events and educational workshops. These initiatives supported improvements in both infrastructure and the educational programs available to children. The collaborative efforts led to increased enrollment rates and improved attendance, showing the direct impact of engaging stakeholders on the program’s success. Conversely, in Kebbi, stakeholder collaboration in ECCDE was less dynamic. Parental and community involvement primarily consisted of attendance at meetings and school events without significant participation in decision-making or school management. This limited engagement resulted in a lesser sense of ownership and had a minor impact on the program’s effectiveness and growth.

In Kebbi, the collaboration between the state Ministry of Environment, local NGOs, and community health workers fostered a comprehensive approach to environmental sanitation. This collaboration facilitated the implementation of sustainable waste management practices and public health campaigns. Although attempts were made to foster stakeholder collaboration in Anambra’s environmental sanitation initiatives, these efforts were often undermined by inconsistent engagement from local government officials and sporadic participation from NGOs. Without a cohesive framework for collaboration, many attempts were short-lived and failed to achieve their intended impact on environmental cleanliness.

In Anambra, a collaboration involving vocational training centers, local industries, and the state Ministry of Labor and Employment led to well-structured skills training programs that were directly aligned with the economic needs of the area. Local businesses and industry leaders were actively involved in designing the curriculum and providing apprenticeship opportunities for trainees. This close partnership ensured that the skills taught were relevant to market demands, facilitating a smoother transition for trainees into gainful employment. In contrast, Kebbi’s skills acquisition programs struggled due to a lack of strong collaboration with local industries. As a result, the training provided was often not tailored to the immediate needs of the local economy. This misalignment reduced the effectiveness of the programs, leading to lower job placement rates and unsatisfactory long-term employment outcomes for participants.

#### Adequate resource allocation

Effective resource allocation is crucial to the success and sustainability of development programs. ATASP-1’s success in both states was significantly supported by sufficient resource allocation. In Kebbi, resources were effectively used to improve irrigation systems and road infrastructure, which are crucial for agricultural activities. In Anambra, resources helped provide farmers with high-quality seeds and training, although some funding delays occasionally impacted the pace of project execution.

The ECCDE program in Anambra has benefitted from innovative resource utilization, including community contributions and partnerships with religious bodies. These efforts compensated for government funding shortfalls and maintained infrastructure and educational quality. In contrast, ECCDE in Kebbi suffered from inadequate resource allocation, which led to high teacher-to-pupil ratios and insufficient caregiver support. The lack of resources hindered the program’s ability to provide quality education and adequately address children’s nutritional and developmental needs.

The availability of resources for environmental sanitation in Kebbi supports the Sanitation Task Force and the sanitation campaigns that contributed to healthier living conditions and reduced diseases associated with poor sanitation. Conversely, resource challenges in Anambra led to inadequate staffing and the outsourcing of essential tasks, compromising the effectiveness of environmental sanitation efforts.

The skills acquisition program in Anambra effectively used resources, including comprehensive training sessions and practical experience opportunities. This thorough approach helped participants gain relevant skills aligned with local economic needs, enhancing their employability. However, in Kebbi, the skills acquisition programs’ community engagement led to more participants but was not supported by additional funding. The insufficient resources led to inadequate training and less effective skill development among participants.

#### Insufficient infrastructure

In Kebbi, the ATASP-1 program faced challenges with infrastructure that were pivotal to agricultural success, such as inadequate road networks and irrigation systems. These deficiencies hindered the transportation of agricultural products to market areas. They were also some challenges the program sought to solve. Similar infrastructure issues were noted in Anambra State, particularly in the delay of constructing essential facilities like storage units and processing plants. These delays were attributed to bureaucratic inefficiencies and funding shortfalls, which slowed the value chain development and impacted the overall program outcomes.

The ECCDE program in Anambra state struggled with insufficient infrastructure, such as inadequate classroom space and lack of proper sanitation facilities. Community contributions and partnerships somewhat mitigated these issues but still impacted the quality of education and school health standards. The situation was more severe in Kebbi, with a noticeable deficiency in the basic educational infrastructure. Schools lacked adequate classrooms, furniture, and learning materials, severely impacting children’s learning environment and educational outcomes.

The environmental sanitation program in Anambra State suffered from a critical lack of infrastructure for effective waste managements, such as adequately designated waste disposal sites and recycling facilities. This led to improper waste handling and increased environmental pollution, exacerbating public health risks. Anambra’s high population density made the lack of infrastructure even more critical. Although efforts were made to improve sanitation infrastructure in Kebbi state, such as establishing a Sanitation Task Force, the lack of continuous funding and maintenance led to the suboptimal operation of sanitation services, which failed to meet the community’s needs effectively.

The lack of adequately equipped training facilities impeded skills acquisition programs in Anambra State. Though some efforts were made to provide necessary resources, there was inconsistency in the availability and quality of training materials. The lack of infrastructure was even more pronounced in Kebbi State, with many training programs operating without the necessary equipment or proper facilities and focusing mostly on theory.

#### Lack of trained personnel

Due to the cultural context of Kebbi State, the ATASP-1 program faced setbacks because of the unavailability of trained female agricultural extension workers and technical experts needed to effectively support farmers in Kebbi. This gap limited the dissemination of innovative farming techniques and slowed the adoption of improved agricultural practices, ultimately affecting productivity and economic outcomes. This challenge was less visible in Anambra State.

A shortage of qualified ECCDE teachers in Anambra hindered the program. The lack of trained educators affected the quality of education and development programs offered to children, compromising educational outcomes and the overall effectiveness of the ECCDE initiative. In Kebbi state, the deficiency in trained personnel was even more acute, with many ECCDE centers operating without enough qualified teachers, impacting the program’s reach and the quality of education provided to young children.

Insufficiently trained environmental health officers compromised environmental sanitation efforts. This lack of specialized personnel impeded the effective implementation of sanitation policies and practices, reducing the program’s ability to improve public health outcomes. In Kebbi state, although efforts were made to address sanitation needs, the lack of trained personnel in sufficient numbers continued to challenge the effective execution of environmental health programs.

The impact of insufficiently trained instructors was evident in the skills acquisition programs in Anambra State, where the quality of training suffered due to the trainers’ inadequate skills and knowledge. The shortage of skilled trainers was a significant barrier in Kebbi, with many training programs being unable to provide high-quality education and practical skills.

#### Bureaucratic inefficiencies

Both states’ bureaucratic inefficiencies manifested in the slow processing of funds and approvals for ATASP-1 projects. This led to delays in constructing infrastructures for agricultural development, such as roads and irrigation systems. These delays affected not only the timeline of the projects but also the seasonal agricultural activities that depended on timely infrastructure improvements.

In Anambra state, bureaucratic delays in funding disbursements and policy implementations impacted the ECCDE program’s ability to upgrade educational facilities and resources timely. Similar bureaucratic hurdles led to prolonged periods without essential resources and support in ECCDE centres in Kebbi state.

In Anambra State, the environmental sanitation efforts were particularly affected by bureaucratic inefficiencies, with delays in funds for waste management infrastructure and the slow response of government bodies to sanitation crises. This lack of timely action exacerbated public health risks and hindered the program’s ability to respond effectively to environmental challenges. In Kebbi, there were fewer bureaucratic inefficiencies in Environmental Sanitation.

Inefficiencies in administrative processes affected the rollout of training programs and the distribution of educational materials in the skills acquisition program at Anambra State. These delays impacted the program’s ability to provide timely and relevant training. Bureaucratic inefficiencies also affected the skills acquisition programs in Kebbi State, where slow certification processes and irregular monitoring of training quality compromised the program’s success.

## Discussion

Building on the operational analysis, this section positions our findings in the broader implementation science and nutrition-sensitive literature, focusing on the mechanisms that cut across sectors and contexts. This study uncovered implementation factors and ensuing areas for individual program improvement (Table 5), showing the complexity of translating and implementing large-scale programs in the real world [22,23]. This table shows that improvement is needed in all programs, thus shifting the discussion from what is delivered to how it is delivered – the operational facilitators and barriers that determine whether planned inputs translate to sustained use.

**Table 5.** Improvement areas of assessed programs: dose, reach, fidelity, and recruitment.

Program	State	Dose	Reach	Fidelity	Recruitment
Agricultural Transformation Agenda Support Program 1 (ATASP1)	Federal/Both States	Prompt release and distribution of ATASP equipment to farmers	Targeted gender-sensitive nutrition training sessions for male and female farmers	N/A	Expand participation through gender-sensitive recruitment strategies
	Anambra	Regular nutrition training for farmers, covering IYCF and micronutrient practices	Partnerships with offtakers to ensure all farm produce is utilized	N/A	N/A
	Kebbi	Improve farmers’ access to anchor borrowers and Bank of Industry loans	N/A	N/A	N/A
Early Childhood Development Education (ECCDE)	Federal/Both States	Continuous training sessions provided for ECCDE teachers through the National Teachers Institute	Increase parental motivation and awareness for ECCDE through interprogram collaboration	Revitalize the National Policy for IECD, covering education, health, nutrition, and child protection	Conduct recruitment drives in high-inequity areas using community influencers, religious institutions, and town criers
	Anambra	Regular PTA meetings with integrated nutrition training	Community investment and support for ECCDE through fundraisers, partnerships, and business collaborations	Establish quality assurance monitoring for ECCDE in schools to ensure adherence to standards	N/A
	Kebbi	Annual professional development sessions for ECCDE teachers	Increase parental nutrition-related training sessions in communities to improve child health	Regular monitoring and quality checks for teacher engagement and participation in training programs	Aggressive recruitment of pupils through announcements in religious centers and by influential community members
Environmental Sanitation	Federal/Both States	State-wide campaigns and sessions to increase awareness of sanitation importance	School clubs and community programs utilized to spread sanitation messages	Improve consistency in implementation through regular performance evaluations of environmental health officers	N/A
	Anambra	Production of IEC materials for sanitation campaigns yearly	Divide the state into sanitation zones, each assigned dedicated staff for effective reach	Equip sanitation departments with vehicles, motorcycles, and other resources for routine inspections	N/A
	Kebbi	Employ more female environmental health officers to conduct sanitation programs	N/A	Convert state Environmental Task Force into a Technical Committee to align with the National Policy on Environmental Sanitation	N/A
Social Welfare	Federal/Both States	Accredited modules integrated with business management and financial education for skills acquisition	Generate awareness of the skills program and recruit participants through community outreach strategies	Strict adherence to established indicators in program evaluation and adaptation	Target recruitment of unemployed youth, women, and vulnerable individuals
	Anambra	Establish skills acquisition centers across all LGAs	N/A	Regular in-service training for program staff to maintain quality and update techniques	N/A
	Kebbi	Conduct post-graduation follow-ups with participants to ensure skills utilization and transition to employment	N/A	N/A	N/A

The identified facilitators across these programs significantly contribute to their outcomes. Community Engagement and Stakeholder Collaboration align program objectives with community needs and expectations, ensuring higher acceptance and sustainability [24–27]. Strong community engagement can facilitate the sustainability of outcomes by fostering local ownership, building trust between implementers and the community, and mobilizing local resources and knowledge for program support [28,29]. Effective community engagement often involves strategies, including regular community meetings, participatory planning sessions, and inclusive decision-making processes [29]. These efforts ensure that programs are receptive to community needs and adaptable to changing local dynamics [30]. Stakeholder collaboration is essential for harnessing a wide array of expertise and perspectives to address complex development challenges effectively [31]. Effective stakeholder collaboration facilitates resource pooling, reduces efforts redundancy, ensures consistency in program implementation, and helps scale successful initiatives [32]. It also enhances the program’s credibility and broader community acceptance by involving diverse voices in the planning and execution phases. For instance, in ATASP-1, the inclusion of farmer feedback in decision-making processes led to interventions more suited to their specific agricultural needs, resulting in better adoption rates and enhanced agricultural productivity. This is similar to the Indian context, where only strengthened local institutions led to improved agriculture-nutrition links [24]. Ensuring that programs are well resourced is also crucial [33]. It provides the financial, material, and human resources required for optimal operation. Adequate resources ensure that programs consistently deliver high-quality services, reach their targeted populations, and effectively achieve desired outcomes. In the context of ECCDE, innovative resource utilization, including community contributions in Anambra, helped maintain the quality of education despite funding shortfalls. This approach ensured the program’s continuity and effectiveness, directly impacting educational outcomes.

Just as facilitators support program success, barriers can critically undermine their effectiveness, suggesting areas for strategic improvement. Insufficient infrastructure and lack of trained personnel are significant constraints that limit program reach and quality [34–39]. Insufficient infrastructure can manifest as inadequate physical facilities, a lack of essential equipment, and poor maintenance, which are crucial for successfully implementing any program [40,41]. Lack of trained personnel involves a shortage of individuals and a deficit in the skills and knowledge required to execute and sustain program initiatives efficiently [40]. In ECCDE, the shortage of qualified teachers due to inadequate training facilities directly affected the educational services provided, mirroring the challenge seen in Skills Acquisition programs where insufficient infrastructure curtailed the quality of training. Bureaucratic Inefficiencies often lead to delays in resource allocation and program execution, as seen in the ATASP-1 and Environmental Sanitation programs. Such inefficiencies slow the implementation process and diminish the programs’ potential impact [42,43]. These inefficiencies can delay the distribution of resources, hinder timely project implementation, and often lead to missed opportunities for program improvement and community impact [43]. Streamlining these processes and enhancing organizational efficiency are crucial to effectively delivering program benefits.

In our analysis, ATASP-1’s success was closely tied to robust stakeholder engagement and adequate resource allocation. This mirrors findings from Kenya, where stakeholder involvement has a positive influence on the completion of government-funded agricultural projects in arid and semi-arid areas in the study area [44]. Our findings in ECCDE emphasized the critical role of community engagement and resource allocation. Comparatively, in Brazil’s well-documented Criança Feliz program, similar community-driven approaches facilitated significant improvements in child development outcomes [45]. Both programs demonstrate that active parental involvement can mitigate government funding shortfalls, yet the necessity of adequate training for ECCDE personnel remains a shared challenge. This suggests that despite different socio-economic conditions, the core elements of successful ECCDE programs – community involvement and educational quality – are universally applicable.

The Environmental Sanitation program in Kebbi benefited from structured community engagement and government support, contrasting sharply with Anambra, where resource limitations and bureaucratic inefficiencies hindered effective implementation. Different studies in Ethiopia have shown that community engagement and community-led WASH programs have WASH practices and reduced diarrheal in the study population [45–47]. In our study, Skills Acquisition Programs in Anambra were more successful due to better resource allocation and linkage with local economic needs, unlike in Kebbi, where training misalignment with market demands was evident. This is consistent with findings from the Technical and Vocational Education and Training (TVET) programs in Kenya, which thrived under similar conditions of adequate funding that provided modern teaching equipment and alignment with industry needs [46]. Both contexts highlight the importance of aligning skills training with local labor market demands to enhance employability and economic independence.

While the preceding section catalogues barriers, some underlying ‘enabling conditions’ cut across sectors and states – community legitimacy and adaptive management. They do not substitute for basic inputs such as trained personnel and functioning infrastructure; rather, they determine how effectively such inputs are mobilized and sustained. Across all four interventions, the depth of community involvement, not merely meeting attendance, explained most of the variance in sustained participation. This aligns with evidence from participatory agricultural extension and community-led WASH [47,48]. Where local actors perceived genuine decisionspace (e.g. the ATASP1 farmer forums in Kebbi), uptake and maintenance were markedly higher. Interventions that institutionalized short ‘senseandrespond’ cycles – quarterly reflection meetings in ATASP1; termly ECCDE quality assurance visits in Anambra – were able to correct course when logistics or participation faltered. Comparable benefits of adaptive management have been proposed in health systems [49].

While our data come from two Nigerian states, the mechanisms we observed are not uniquely Nigerian. They reflect broader implementation dynamics that shape nutrition-sensitive delivery wherever interventions are grafted onto preexisting sectoral systems. The following points, therefore, speak to policy actors in similarly resource-constrained, decentralized contexts. Embedding nutrition-specific indicators into sector performance contracts can guard against mandate drift. Investing in legitimacy is crucial, as budget lines for participatory planning, local leadership training, and transparent feedback loops routinely deliver higher returns in reach and fidelity than equivalent investments in physical assets [50,51]. Adaptive management and the reflection process are essential to course-correct early and should be institutionalized.

This study contributes valuable insights into implementing nutrition-sensitive programs in Anambra and Kebbi states, utilizing qualitative methods to capture complex impact pathways. Its detailed examination of contextual factors influencing program success is a key strength. However, limitations include the study’s reliance on qualitative data, which may not comprehensively capture all program impact dimensions.

## Conclusion

This multisector analysis confirms that nutrition-sensitive programs succeed when enabling conditions align: community legitimacy that guarantees genuine reach and governance structures that permit rapid adaptation. When any link is weak, even well-resourced programs underdeliver. They must, however, be paired with adequate resources, skilled staff, and streamlined procedures if nutrition-sensitive investments are to achieve their full potential.

For policymakers in Anambra and Kebbi – and in similar decentralized contexts – the immediate priority is to translate these enabling conditions into practice: embed nutrition indicators in sector work plans, invest in participatory implementation to build local legitimacy, and institutionalize quarterly learning cycles. Future research should test the transferability of these mechanisms across settings and program types, accelerating the evidence base for nutrition-sensitive scale-up.

## Supplementary Material

Supplemental Material

## Data Availability

Raw data, both print and electronic, and the dissertation are being stored at the Division of Human Nutrition at Stellenbosch University. The data can be made available by request to the corresponding author.
