# Supplementary material for: Implementation matters: program impact pathway analysis of four sectoral nutrition-sensitive interventions in Anambra and Kebbi states, Nigeria
Source: Glob Health Action. 2025 Jun 26;18(1):2519677. doi: 10.1080/16549716.2025.2519677 (PMC12203689; doi:10.1080/16549716.2025.2519677)
Supplement: Supplemental Material [file ZGHA_A_2519677_SM7101.docx]

**Supplementary materials**

**Operational analysis guide**

Name of programme: ………………………………. Programme no.: ……………..

Ministry in charge of the programme: ……………………

- - 1. Does specific implementation or operations plans exist for the programme? Probe for:
       - Implementation authority
       - Programme content
       - Implementation support
       - Perceived pathway to a nutrition outcome *(Is there any document to support this?)*
       - What is the expected outcome of the programme?
       - Does it impact on children’s nutritional status?
    2. What are the institutional arrangements for delivery of the programme? Probe for:
       - Service delivery at different levels
       - Timing of programme
    3. What are institutional arrangements for training? Probe for:
       - Specific activities for training
       - Scope of training
       - Availability of training manual
    4. Are monitoring and evaluation mechanisms included in the programme design? Probe for:
       - The exact mechanisms

**In-depth interview guide for service delivery workers**

Name of programme: ………………………………. Programme no.: ……………..

Ministry in charge of the programme: ……………………

Interview number: ………………..

Age of interviewee: ……………….

Education: ………………

Gender: ………………

**Section A: Management and support services**

- - - 1. How do programme managers at the state secretariat and the ministry support the planned activities to enable delivery of results?

- What do you think is the aim of your programme?

- Do you think your programme is achieving that aim?

- Do the programme managers at the state secretariat help?

- How do they help?

- - - 1. Do supervision and management occur as planned? To what extent?

- Who are your direct supervisors at the ministry?

- Do they visit here in a supervisory role?

- How often does that happen?

- How often are management meetings held?

- Who attends the meetings?

**Section B: Training and capacity development**

1. To what extent has training and capacity building been implemented?

- How many training sessions have you and other staff attended in the recent past?

- How often are these training sessions held?

- What are the contents of those training sessions you attended?

- Do you think they have helped?

- How can they be made better?

1. Who conducted the training sessions and who participated?

- Who oversaw the training sessions?

- Where were they held?

- Who were the participants?

**Section C: Service delivery fidelity**

1. Workers’ workload

– Do you have sufficient workers?

– Do you think the workload is more than they can handle?

1. Demand for nutrition services

– Are the services provided accessed by citizens?

–- What is the estimated rate of service uptake?

1. Equipment and work aid

- What equipment do you have here?

- Is it sufficient?

- Apart from what you listed, are there other work aids that are provided for you?

- What other equipment or work aids would make this programme function better?

**Exit interview guide for service delivery beneficiaries**

**Instructions**: Use the guide below to interview a program beneficiaries

Name of programme: ………………………………. Programme no.: ……………..

Ministry in charge of the programme: ……………………

Interview no.: ………………..

Age of interviewee: ………………..

Education: ………………..

Gender: ………………..

1. What is the level of different programme components to the users met at the service delivery point? *Probe for:*

- What programme did you come for?

- Did you get the service you wanted?

- Were you well attended to?

1. How often do they utilise the programme?
2. What are their perceived benefits? *Probe for:*

- Are you happy with what you gained from the programme?

- Reasons for satisfaction or lack of it
